# Characterization of a Human Sapovirus Genotype GII.3 Strain Generated by a Reverse Genetics System: VP2 Is a Minor Structural Protein of the Virion

**DOI:** 10.3390/v14081649

**Published:** 2022-07-27

**Authors:** Tian-Cheng Li, Michiyo Kataoka, Yen Hai Doan, Hiroyuki Saito, Hirotaka Takagi, Masamichi Muramatsu, Tomoichiro Oka

**Affiliations:** 1 Department of Virology II, National Institute of Infectious Diseases, Tokyo 208-0011, Japan; muramatsu@niid.go.jp; 2 Department of Pathology, National Institute of Infectious Diseases, Tokyo 208-0011, Japan; michiyo@niid.go.jp; 3 Center for Emergency Preparedness and Response, National Institute of Infectious Diseases, Tokyo 208-0011, Japan; yendoan@niid.go.jp; 4 Akita Prefectural Research Center for Public Health and Environment, Akita 010-0874, Japan; hrsaito@akita.office.ne.jp; 5 Management Department of Biosafety, Laboratory Animal and Pathogen Bank, National Institute of Infectious Diseases, Tokyo 208-0011, Japan; htakagi@niid.go.jp

**Keywords:** human sapovirus, HuSaV, genogroup GII.3, virus particle, reverse genetics system, VP1, VP2

## Abstract

We devised a reverse genetics system to generate an infectious human sapovirus (HuSaV) GII.3 virus. Capped/uncapped full-length RNAs derived from HuSaV GII.3 AK11 strain generated by in vitro transcription were used to transfect HuTu80 human duodenum carcinoma cells; infectious viruses were recovered from the capped RNA-transfected cells and passaged in the cells. Genome-wide analyses indicated no nucleotide sequence change in the virus genomes in the cell-culture supernatants recovered from the transfection or those from the subsequent infection. No virus growth was detected in the uncapped RNA-transfected cells, suggesting that the 5′-cap structure is essential for the virus’ generation and replication. Two types of virus particles were purified from the cell-culture supernatant. The complete particles were 39.2-nm-dia., at 1.350 g/cm^3^ density; the empty particles were 42.2-nm-dia. at 1.286 g/cm^3^. Two proteins (58-kDa p58 and 17-kDa p17) were detected from the purified particles; their molecular weight were similar to those of VP1 (~60-kDa) and VP2 (~16-kDa) of AK11 strain deduced from their amino acids (aa) sequences. Protein p58 interacted with HuSaV GII.3-VP1-specific antiserum, suggesting that p58 is HuSaV VP1. A total of 94 (57%) aa of p17 were identified by mass spectrometry; the sequences were identical to those of VP2, indicating that the p17 is the VP2 of AK11. Our new method produced infectious HuSaVs and demonstrated that VP2 is the minor protein of the virion, suggested to be involved in the HuSaV assembly.

## 1. Introduction

Sapovirus (SaV) is a positive-sense single-stranded RNA virus that belongs to the genus *Sapovirus* in the family *Caliciviridae* [1]. SaVs have been detected in humans and other animals including pigs, mink, dogs, rats, sea lions, bats, and chimpanzees [2]. There are 19 genogroups of the virus, and four of them (GI, GII, GIV, and GV) were detected from humans [3,4]. The antigenicity differs not only among the genogroups but also among the genotypes within the particular genogroups, such as GI and GII [5,6,7]. Human SaVs (HuSaVs) are responsible for both sporadic cases and occasional outbreaks of acute gastroenteritis, and the transmission of HuSaV is thought to occur through fecal-oral routes [1,8,9,10,11,12,13].

The 7.1- to 7.7-kb-long SaV genome contains a short 5′-terminal untranslated region that starts with GTG, two open reading frames (ORFs), and a 3′-terminal untranslated region followed by a poly A tail. ORF1 encodes a polyprotein consisting of ~2280 amino acids (aa), and it is processed into multiple nonstructural (NS) proteins (NS1, NS2, NS3, NS4, NS5 [VPg] and NS6-NS7) and a structural protein (VP1) by a virus-encoded protease, NS6 [1,2,14,15]. A subgenomic RNA was identified during the replication of a porcine SaV (PoSaV) [16]. The subgenomic RNA of the HuSaV GI.1 Manchester strain (GenBank accession no. X86560) consists of ~2300 nucleotides (nt) and starts with GTG (nt 5161 to 5163) and terminates at the end of the genome [1,17].

Thus, two mechanisms may underlie the production of SaV VP1. One is that VP1 is cleaved from the ORF1-encoded polyprotein, and the other is that VP1 is translated from the subgenomic RNA [18]. VP1 consists of ~560 aa and is the major capsid component of the SaV virion; the molecular weight (MW) of the PoSaV VP1 is ~58 kDa [19]. The expression of sapovirus VP1 has been achieved with the use of insect or mammalian cells, followed by a spontaneous assembly of VP1 forming virus-like particles (VLPs), which are morphologically similar to native SaV, which has been demonstrated [5,6,7,20,21,22]. ORF2 is predicted to encode a minor ~17-kDa structural protein, VP2. Although PoSaV VP2 was detected in in vitro translation products of a full-length genomic cDNA, it was not identified in porcine SaV virions [19,23]. The function of VP2 is thus unclear.

A reverse genetic system is a powerful tool that is widely used to produce infectious viruses from cloned cDNA. Although this system worked in the case of the PoSaV [23], no infectious HuSaV viruses have been recovered with a reverse genetics system. The lack of a cell culture system for the propagation of human SaVs (HuSaV) has so far prevented their rescue by a reverse genetics technique [24]. Recently, a human duodenum carcinoma cell line, HuTu80, was established and shown to propagate HuSaVs of the genotype 3 of genogroup 2 (GII.3), GI.1 and GI.2 strains [25], providing a promising system for the propagation of HuSaV.

In the present study, we generated an infectious HuSaV GII.3 using a reverse genetics system with HuTu80 cells, and we confirmed that VP1 and VP2 were the major and minor structural proteins of the virion. These results were further confirmed by experiments using a HuSaV GI.1 strain.

## 2. Materials and Methods

### 2.1. In Vitro Transcription of HuSaV GII.3 RNA

**Figure 1:**
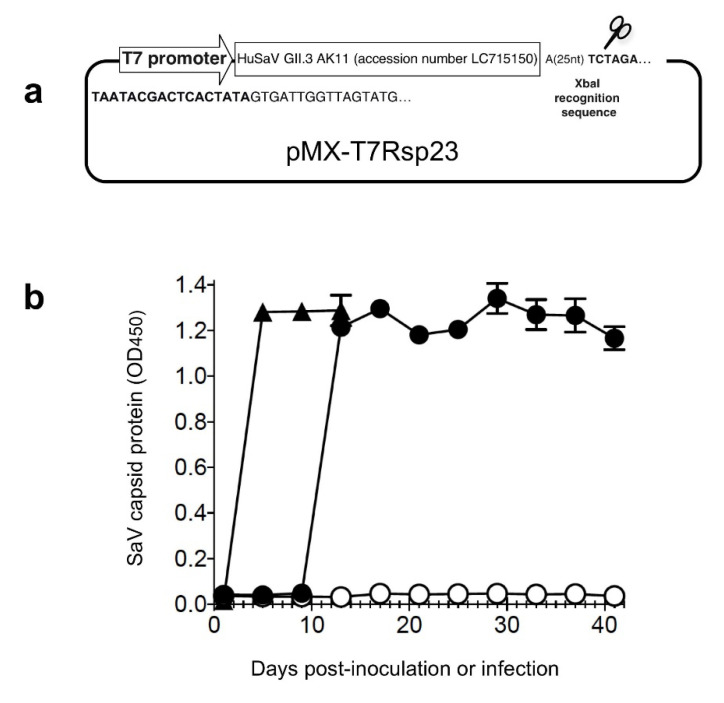
Generation and replication of HuSaV GII.3 AK11 strain in HuTu80 cells. A schematic diagram of the plasmid pMX-T7Rsp23 is shown (**a**). HuTu80 cells were transfected with either capped (⬤) or uncapped (⭘) full-length AK11 RNA, and the culture supernatant (p0) was collected every 4 days and used for detection of the capsid protein by an antigen-capture ELISA (**b**). The supernatant p0 collected on day 16 p.i. from the capped RNA-transfected cells was used to inoculate HuTu80 cells, and the capsid protein in the cell culture supernatant (p1) was examined similarly (▲) (**b**). Triplicate samples were used for transfection and inoculation. The mean OD value is shown and the error bars indicate the SD.

A complete genome of HuSaV GII.3 AK11 strain [25] was synthesized based on the nt sequence deposited in GenBank (accession no. LC715150), to which a T7 RNA polymerase promoter sequence was added at the 5′ end, and an *Xba*I site was added at the 3′ end following a poly A tail (25 nt). This construct was cloned into a pMX vector to generate a plasmid, pMX-T7Rsp23 (Thermo Fisher Scientific, Waltham, MA, USA) (Figure 1a). The plasmid was linearized with *Xba*I and purified by phenol/chloroform extraction. Capped and uncapped RNAs were synthesized using an mMACHINE T7 kit and a MEGAscript kit (Ambion, Austin, TX, USA), respectively. All RNAs were purified by lithium chloride precipitation, as described [26].

### 2.2. Cell Culture, Transfection, and Virus Inoculation

A human duodenum carcinoma cell line, HuTu80 (American Type Culture Collection [ATCC] HTB-40^TM^), was grown in Iscove’s modified Dulbecco’s medium (IMDM) (Sigma-Aldrich, St. Louis, MO, USA) supplemented with glutamine (Gibco, Grand Island, NY, USA), 5% (*v*/*v*) heat-inactivated fetal bovine serum (FBS) (Biosera, Kansas City, MO, USA), 100 U/mL penicillin, and 100 µg/mL streptomycin (Gibco) at 37 °C in a humidified 5% CO_2_ atmosphere, and passaged every 7 days [25].

The confluent HuTu80 cells were dispersed by trypsinization, and 2 × 10^5^ cells were cultured in a 25-cm^2^ tissue culture flask for 24 h. The cells were then washed with Dulbecco’s phosphate buffered saline (PBS) without Mg^2+^ and Ca^2+^ [PBS (−)] and maintained with 6.6 mL of maintenance medium containing IMDM supplemented with 3% (*v*/*v*) heat-inactivated FBS and 1 mM glycocholic acid sodium salt (Nacalai Tesque, Kyoto, Japan). Transfection was performed using a *Trans*IT-mRNA Transfection Kit (Mirus Bio, Madison, WI, USA), and each transfection was repeated three times. Briefly, 6.6 µg of the capped or uncapped HuSaV GII.3 RNA was combined with 650 µL of Opti-MEM (Gibco), and then 13.2 µL of mRNA boost reagent and 13.2 µL of *Trans*IT-mRNA reagent were added to the mixture. After 5 min of incubation at room temperature (RT), the mixture was added to the HuTu80 cells. After a 12-h incubation at 37 °C, the medium was replaced with 10 mL of the maintenance medium, followed by further incubation at 37 °C. The medium was replaced with the maintenance medium every 4 days, and the culture supernatant was used for the detection of the capsid protein.

For the virus inoculation, the confluent HuTu80 cells were trypsinized, and 2 × 10^5^ cells were cultured in a 25-cm^2^ tissue culture flask. After incubation for 24 h, the cells were washed two times with PBS (−), and a total of 1 mL of the HuSaV GII.3-positive cell culture supernatant was inoculated onto the HuTu80 cells. After adsorption at 37 °C for 1 h, the cells were washed three times with PBS (−), and replaced with 10 mL of the maintenance medium, followed by further incubation at 37 °C.

### 2.3. Antigen Enzyme-Linked Immunosorbent Assay (ELISA) for the Detection of HuSaV GII.3 Capsid VP1 Protein

The capsid VP1 protein in the cell culture supernatant was detected using an antigen detection ELISA, as described [25]. Briefly, flat-bottom 96-well polystyrene microplates (Immulon 2 HB, Dynex Technologies, Chantilly, VA, USA) were coated with 50 µL per well of the rabbit hyperimmune antiserum against HuSaV GII.3 C12 VP1–VLPs at 1:5000 dilution in 0.05 M carbonate buffer (pH 9.6) [7]. The plates were incubated overnight at 4 °C, washed twice with PBS (−), and then blocked with 250 µL of PBS (−) containing 0.5% casein for 2 h at RT or overnight at 4 °C. After the wells were washed three times with PBS (−) containing 0.1% Tween 20 (PBS-T), 50 µL of the cell culture supernatants was added. The plates were incubated for 1 h at RT.

The detection of the capsid VP1 protein was performed using guinea pig hyperimmune antiserum against HuSaV GII.3 C12 VP1–VLPs [7]. After the wells were washed three times with PBS-T, 50 µL of the hyperimmune serum (1:3000) was added to each well. The plates were incubated for 1 h at RT followed by three washes with PBS-T. Next, 50 µL of horseradish peroxidase-conjugated goat anti-guinea pig IgG (IgG H+L) (Rockland Immunochemicals, Philadelphia, PA, USA) at 1:4000 dilution in PBS-T containing 0.25% casein was added to each well. The plates were incubated for 1 h at RT and then washed three times with PBS-T.

Finally, 50 µL per well of 1 mM substrate 3, 3′, 5, 5′-tetramethylbenzidine (Sigma-Aldrich) and 0.01% H_2_O_2_ in citrate buffer (pH 3.5) was added, and the plates were left in the dark for 30 min at RT. The reaction was stopped by the addition of 50 µL per well of 1 M H_2_SO_4_, and the absorbance was measured at 450 nm using a Benchmark Plus Microplate Reader (Bio-Rad Laboratories, Hercules, CA, USA). The absorbance at 750 nm was used as the reference for background subtraction. The uninfected HuTu80 cell culture supernatant (three wells per plate) served as the negative control. When the ratio of the optical density (OD) values between the sample and negative control was >3.0, the sample was judged to be positive.

### 2.4. Real-Time Reverse Transcription-Quantitative Polymerase Chain Reaction (RT-qPCR) for the Detection of HuSaV GII.3 RNA

The viral RNA was extracted from 100 µL of the samples with the use of a High PureRNA Isolation Kit (Roche Applied Science, Mannheim, Germany) according to the manufacturer’s recommendations. The cDNA was synthesized using a random hexamer (Takara, Shiga, Japan) and a ReverTra Ace (Toyobo, Osaka, Japan) and then quantified by a TaqMan real-time PCR with a 7500 Fast Real Time PCR System (Applied Biosystems, Foster City, CA, USA). The RT-qPCR was performed with a mixed forward primer, i.e., HuSaV-F1 (5′-GGCHCTYGCCACCTAYAAYG-3′), HuSaV-F2 (5′-GACCARGCHCTCGCYACCTAYGA-3′), and HuSaV-F3 (5′-GCWRYKGCWTGYTAYAACAGC-3′); a reverse primer, HuSaV-R (5′-CCYTCCATYTCAAACACTA-3′); a FAM-labeled MGB probe, HuSaV-TP-a (5′-FAM-CCNCCWATRWACCA-MGB-NFQ-3′); and QuantiTect Probe PCR Master Mix (Qiagen, Hilden, Germany) under the following conditions: 95 °C for 15 min, followed by 45 cycles of a two-step PCR: 95 °C for 15 s and 60 °C for 60 s. The amplification data were collected and analyzed with 7500 software v2.0.6 (Applied Biosystems) [27].

### 2.5. Purification of HuSaV Particles

The cell culture supernatant collected from the infected cells was clarified by centrifugation at 10,000× *g* for 30 min, and the supernatant was concentrated by ultracentrifugation at 100,000× *g* for 3 h in a Beckman SW 32 Ti rotor. The resulting pellet was suspended in PBS (−) at 4 °C overnight. For CsCl gradient centrifugation, 4.5 mL of the samples was mixed with 2.1 g of CsCl and centrifuged at 100,000× *g* for 24 h at 10 °C in a Beckman SW55Ti rotor. The gradient was fractionated into 250 μL aliquots, and each fraction was weighed to estimate the buoyant density. Each fraction was diluted with PBS (−) and centrifuged for 2 h at 112,000× *g* in a Beckman TLA55 rotor, and the pellet was resuspended in PBS (−) and used for the detection of the viral RNA and protein.

### 2.6. Sodium Dodecyl Sulfate-Polyacrylamide Gel Electrophoresis (SDS-PAGE) and Western Blot Analysis

The viral proteins in each fraction were separated by 5–20% e-PAGEL (Atto, Tokyo) and then stained with Coomassie brilliant blue (CBB). The proteins in the gel were electrophoretically transferred onto a nitrocellulose membrane. The membrane was then blocked with PBS-T containing 5% skim milk and incubated with a rabbit hyperimmune antiserum against HuSaV GII.3 C12 VP1–VLPs (1:1000) in PBS-T containing 1% skim milk [7]. Detection of the rabbit IgG antibody was achieved using alkaline phosphatase-conjugated goat anti-rabbit antibody (1: 1000) (Chemicon International, Billerica, MA, USA). Nitro blue tetrazolium chloride and 5-bromo-4-chloro-3-indolyl phosphate p-toluidine were used for the detection of antibody binding (Bio-Rad Laboratories).

### 2.7. Transmission Electron Microscopy (TEM)

The purified viral particles were placed on a formvar and carbon-coated grid for 45 s, rinsed with distilled water, and stained with a 2% uranyl acetate solution. The grids were observed under a transmission electron microscope (HT7700; Hitachi High Technologies, Tokyo, Japan) at 80 kV.

### 2.8. Viral Genome Sequencing

The entire genome sequence of the HuSaV GII.3 strain produced by the reverse genetics system was determined by next-generation sequencing (NGS), as described [28]. Briefly, a 200-base pair (bp) fragment library was constructed for each sample with the NEBNext Ultra RNA Library Prep Kit for Illumina ver. 1.2 (New England Biolabs, Ipswich, MA, USA) according to the manufacturer’s instructions. Samples were bar-coded for multiplexing with the use of NEBNext Multiplex Oligos for Illumina and Index Primer Sets 1 and 2 (New England Biolabs). Library purification was done with Agencourt AMPure XP magnetic beads (Beckman Coulter, Pasadena, CA, USA) as recommended in the NEBNext protocol.

The quality of the purified libraries was assessed on an MCE-202 MultiNA Microchip Electrophoresis System (Shimadzu, Kyoto, Japan), and the concentrations were determined on a Qubit 2.0 fluorometer using the Qubit HS DNA Assay (Invitrogen, Carlsbad, CA, USA). A 151-cycle paired-end read sequencing run was carried out on a MiSeq desktop sequencer (Illumina, San Diego, CA) using the MiSeq Reagent Kit ver. 2 (300 cycles). After a preliminary analysis, the MiSeq reporter program was used to generate FASTQ formatted sequence data for each sample. The sequence data were analyzed using CLC Genomics Workbench Software ver. 6.5.1 (CLC Bio, Aarhus, Denmark). Contigs were assembled from the obtained sequence reads by de novo assembly. The assembled contig sequences were subsequently used to query the non-redundant nucleotide database in GenBank with the BLAST algorithm [28].

### 2.9. Mass Spectrometry Analysis

The purified HuSaV particles were separated by SDS-PAGE, and the protein bands were cut out. The aa sequence was analyzed by liquid chromatography-tandem mass spectrometry (LC-MS/MS) by an outside company (Integrale, Tokushima, Japan) using the EASY-nLC1200 system (Thermo Fisher Scientific). The data were collected and analyzed with Mascot Server (available online: https://www.matrixscience.com/help_index.html, accessed on 20 June 2022).

### 2.10. N-Terminal Amino Acid Sequence Analysis

The purified virus particles were separated by SDS-PAGE and transferred to a polyvinylidene difluoride (PVDF) membrane. The proteins were stained with CBB, and the bands identified were cut out. The N-terminal aa microsequencing was carried out at Hokkaido System Sciences (Hokkaido, Japan) using the protein sequencer Procise 494HT (Applied Biosystems).

## 3. Results

### 3.1. Generation of the Infectious HuSaV GII.3 AK11 Strain

The capped RNA encoding the entire genome of HuSaV GII.3 AK11 strain was used to transfect HuTu80 cells, and the culture supernatant (p0) was collected every 4 days and used for the detection of the capsid VP1 protein by an antigen-capture ELISA. The capsid protein was detected on day 12 post-transfection (p.t.), with an OD value of 1.215, and it was constantly detected thereafter with similar OD values until day 40 p.t. (Figure 1). In contrast, no viral capsid protein was detected in the supernatants of the uncapped RNA-transfected cells, even as of day 40 p.t. Although the transfection was repeated two more times, the results indicated that the uncapped RNA was not capable of replicating and generating the infectious virus (Figure 1b).

The supernatant p0 collected on day 16 p.t. was used to inoculate HuTu80 cells, and the culture supernatant (p1) was collected to examine whether the p0 supernatant contained infectious viruses. The OD value of 1.281 was observed on day 4 post-infection (p.i.), and similar values were detected on days 8 and 12 p.i. (Figure 1), indicating that the p0 supernatant was infectious and the capped RNA produced infectious AK11 viruses. No obvious cytopathic effects were observed in the HuSaV GII.3-infected cells. The virus in supernatant p0 was designated HuSaV GII.3 AK11 p0, and the virus in supernatant p1 was designated HuSaV GII.3 AK11 p1.

To determine whether the genetic mutations occurred during the virus replication, we used the AK11 p0 collected on day 28 p.t. and AK11 p1 collected on day 12 p.i. for NGS. The entire nucleotide sequence of both AK11 viruses was identified, with the exception of the first 10 nt on the 5′ terminus, and the identified sequences were identical to that of the original nucleotide sequence of the AK11 strain. These results indicated that (i) the generation of the virus occurred in a cap-dependent manner, and (ii) virus replication occurred efficiently in HuTu80 cells.

### 3.2. Purification and Characterization of the HuSaV GII.3 AK11 Virions

**Figure 2:**
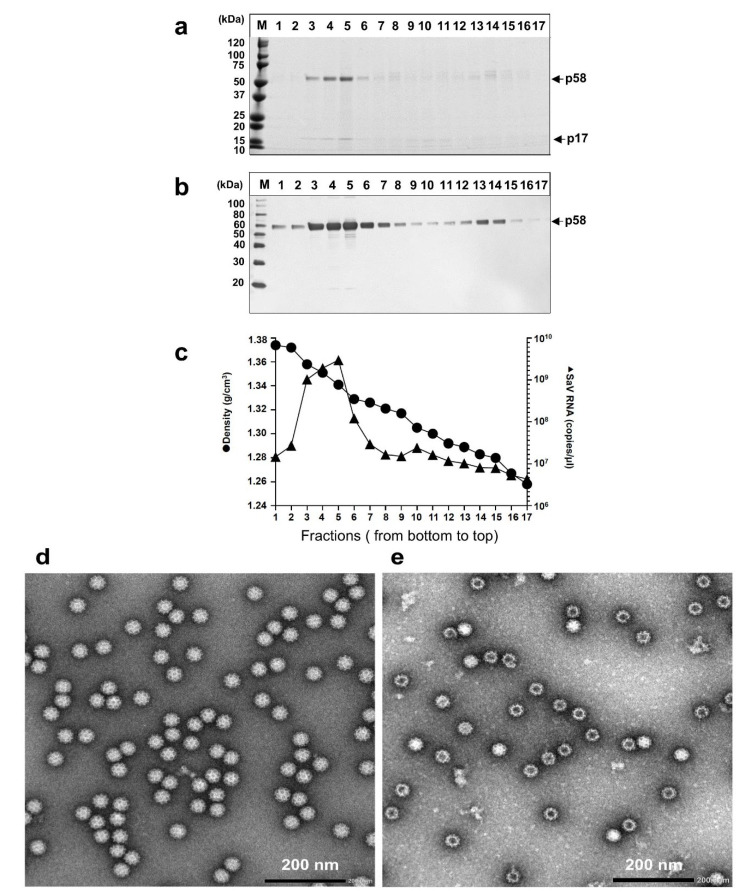
Purification and characterization of AK11 virions. The p1 supernatants collected on day 8 p.i. were concentrated by ultracentrifugation and then purified by CsCl equilibrium density gradient centrifugation. Aliquots from each fraction were analyzed by electrophoresis on 5–20% polyacrylamide gels and stained with CBB (**a**), and the capsid protein was detected by a Western blotting assay using a VP1-specific antiserum (**b**). Molecular weight markers (in kDa) are indicated on the left (**a**,**b**). Viral RNA in each fraction (▲) detected by RT-qPCR and the density (⬤) is shown (**c**). Electron micrographs of fractions 5 (**d**) and 13 (**e**). Bar: 200 nm. The particle sizes were determined using Hitachi EMIP software ver. 0524 (Hitachi High Technologies, Tokyo, Japan).

A total of 300 mL of the p1 supernatant was concentrated and purified by CsCl gradient ultracentrifugation as described in the Section 2. When the pelleted virions were analyzed by SDS-PAGE followed by CBB staining, two protein bands, p58 (58 kDa) and p17 (17 kDa), were observed primarily in fractions 3 to 5 (Figure 2a). The MWs of VP1 and VP2 of AK11 strain based on their aa sequences, ~60 kDa and ~16 kDa, were similar to those of p58 and p17. A Western blot assay indicated that HuSaV GII.3 C12 VP1-specific antiserum is bound to p58 (Figure 2b), demonstrating that p58 is the VP1 of AK11 virus. In addition to fractions 3–5, strong bands of p58 were observed in fractions 13 and 14 by Western blot assay, suggesting that p58 exists with different densities (Figure 2b).

We next examined the viral RNA copy numbers in each fraction by RT-qPCR. A peak of viral RNA was detected in fractions 3, 4, and 5, at the values 1.0 × 10^9^ copies/μL, 1.9 × 10^9^ copies/μL, and 3.0 × 10^9^ copies/μL, respectively (Figure 2c). The mean density of fractions 3, 4, and 5 was 1.350 g/cm^3^ (1.358 g/cm^3^, 1.351 g/cm^3^, and 1.341 g/cm^3^), and that of fractions 13 and 14 was 1.286 g/cm^3^ (1.289 g/cm^3^ and 1.283 g/cm^3^) (Figure 2c).

Electron microscopy revealed many spherical particles showing many cup-shaped depressions in fractions 3, 4, and 5, and the average particle size was 39.2 nm in diameter (*n* = 5, 38.1 to 39.8 nm) (Figure 2d). The virus particles were also observed in fractions 13 and 14, but most of them were empty, and the average size of the empty particles was 42.2 nm dia. (*n* = 5, 41.8 to 42.6 nm) (Figure 2e). These results indicated that two types of virus particles were produced in HuTu80 cells: the complete viral 39.2-nm-dia. particles at the density of 1.350 g/cm^3^ in CsCl and the empty 42.2-nm-dia. particles at the density of 1.286 g/cm^3^ in CsCl. Both types of particles were released into the cell culture supernatants.

### 3.3. Analyses of the Capsid Proteins of AK11 and AK20 Virion

**Figure 3:**
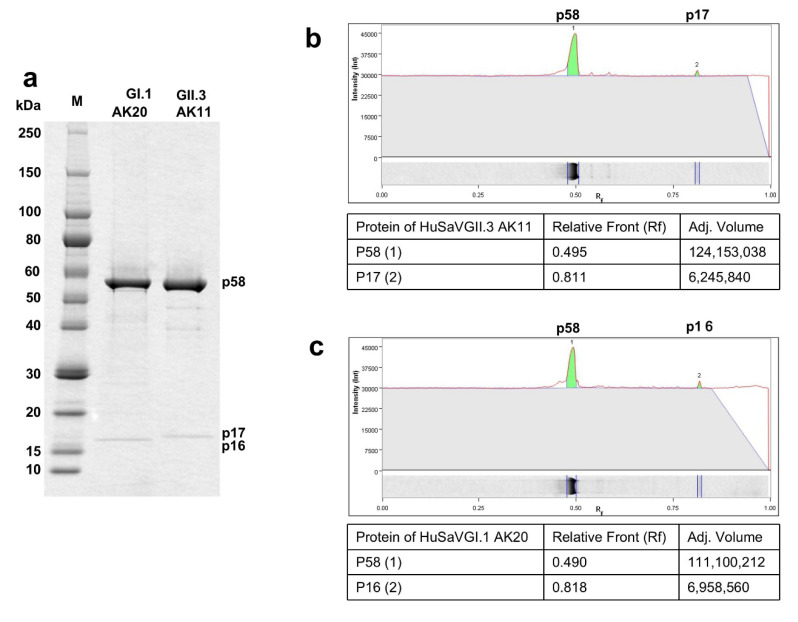

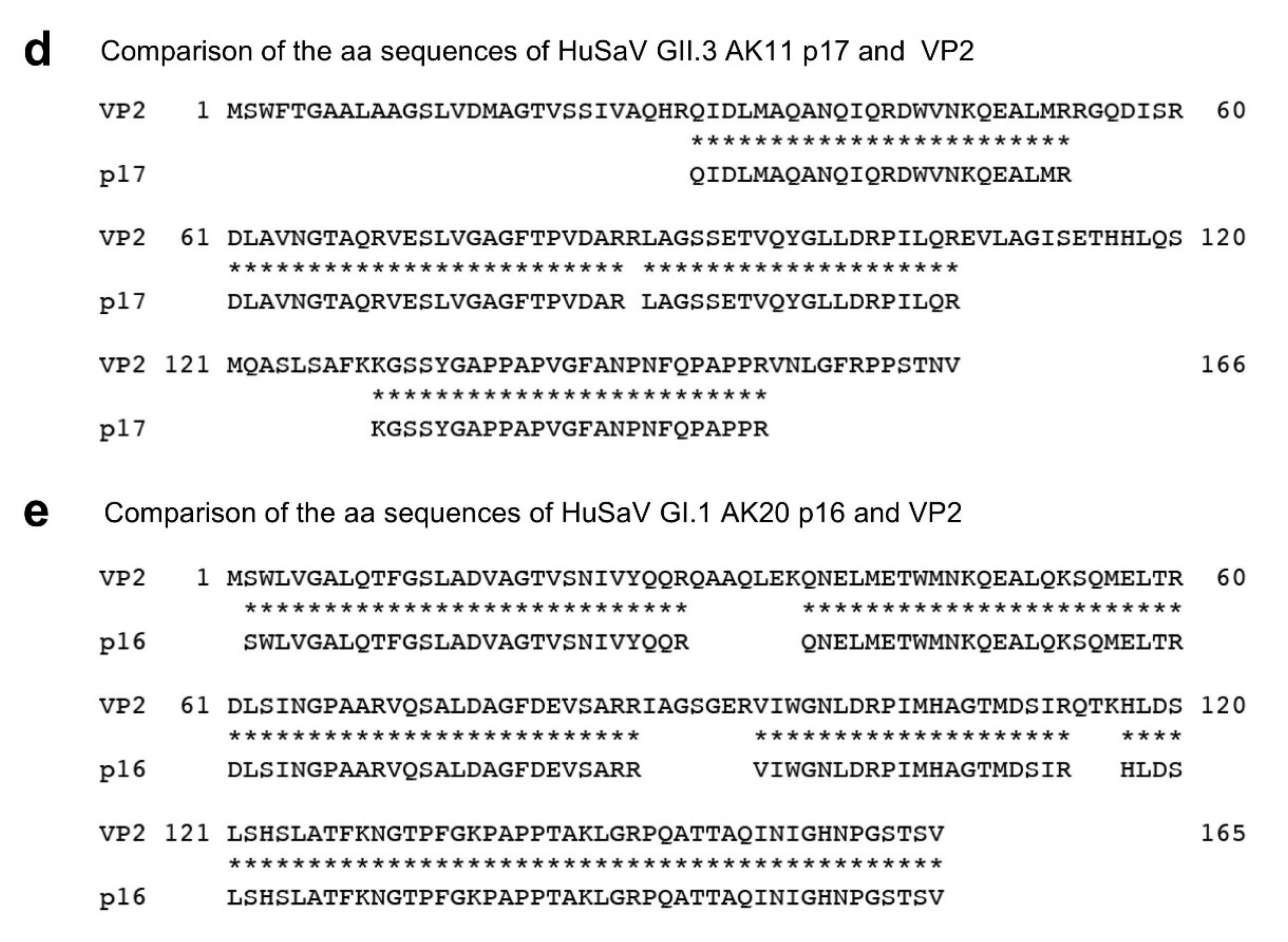
Proteins analyses of the p17 and p16. Purified HuSaV GI.1 AK20 virions and HuSaV GII.3 AK11 virions were analyzed by SDS-PAGE followed by CBB staining (**a**). The ratio of the protein content between p58 and p17 of HuSaV GII.3 AK11 (**b**), and that between p58 and p16 of HuSaV GI.1 AK20 (**c**) were quantitated by Image software ver. 6.1 based on the band intensities. The identified aa sequences of p17 was compared with those of VP2 of HuSaV GII.3 AK11 (**d**), and the aa sequences of p16 was compared with those of VP2 of HuSaV GI.1AK20 (**e**).

VP2 of a sapovirus is thought to be a minor structural protein, but this is controversial. Since we observed that p17 was present in the purified virus particles and its MW was similar to that of VP2 of AK11, we speculate that p17 is highly likely to be VP2. To investigate whether p17 exists in other HuSaV strains, we purified HuSaV GI.1 AK20 (LC715151) (AK20) particles from the cell culture supernatants of infected HuTu80 cells, and we compared the structural proteins with those of AK11 by SDS-PAGE, followed by CBB staining (Figure 3a). In addition to a major band corresponding to p58, we observed a faint 16-kDa band (i.e., p16) in the AK20 particles. When the AK11 virions were analyzed at the same time, we observed a major p58 band and a similar faint 17-kDa band (p17), suggesting that the two small proteins were minor structural proteins of AK20 and AK11 virions, although they belong to different genogroups.

To further examine the functions of these small proteins, we determined the ratio of the protein content between p58 and p16 or p17 using Image Lab^TM^ software ver. 6.1 (Bio-Rad, Hercules, CA, USA) based on the SDS-PAGE image. The results showed that the ratio between p58 and p17 in the HuSaV GII.3 AK11 particles was 20:1 (124,153,036:6,245,840) (Figure 3b) and between p58 and p16 in the HuSaV GI.1 AK20 particles was 16:1 (111,100,212:6,958,560) (Figure 3c). These results demonstrated that p17 and p16 were minor proteins present in the HuSaV particles.

We further analyzed the aa sequences of the p17 and p16 by LC-MS/MS and compared them with the aa sequences of the corresponding VP2. As shown in Figure 3d, VP2 of HuSaV GII.3 AK11 consisted of 166 aa, and a total of 94 aa (57%) sequences derived from p17 corresponded to aa 30–53, 61–85, 87–106, and 130–155. Similarly, VP2 of HuSaV GI.1 AK20 consisted of 165 aa, and a total of 147 aa (89%) derived from p16 corresponded to aa 2–29, 37–86, 94–113 and 117–165 (Figure 3e). These results clearly indicate that the p16 is the VP2 of HuSaV GI.1 AK20 and p17 is the VP2 of HuSaV GII.3 AK11. In other words, VP2 is a component of HuSaV particles. The N-terminal amino acid analyses revealed that the N-termini of p58 of both HuSaVs were blocked.

## 4. Discussion

Although feline calicivirus (FCV) and PoSaV have been produced ex vivo by reverse genetic systems using synthetic RNA [23,29], no HuSaV has been successfully produced, since no cell culture system has been developed for the successive replication of the virus. Calicivirus genomes are modified at their 5′ ends by a covalently linked VPg [18]. In the present study, we synthesized the capped and uncapped genome RNAs of HuSaV GII.3 AK11 strain and used them to transfect HuTu80 cells. An infectious virus was recovered from the capped RNA-transfected cells, whereas the virus was not generated in the uncapped RNA–transfected cells, suggesting that the 5′-end capping of HuSaV RNA compensates the VPg functions and is essential for the production of the infectious viruses, as was observed for PoSaV and FCV [23,29]. Our results confirm the usability of the reverse genetics system to produce infectious HuSaVs.

Caliciviruses have a small positive-sense RNA genome (~7.5 kb) and are packaged within a *T* = 3 icosahedral shell, assembled from 180 copies of the major capsid protein (VP1) in three quasi-equivalent settings [30]. In addition to VP1, an essential but low copy-number of the capsid protein, VP2, which is encoded in all calicivirus genomes, is incorporated in the virion [31]. The VP2 of FCV forms a large portal-like assembly at a unique three-fold axis of symmetry after receptor engagement, and the FCV particle formed with twelve copies of VP2 [31].

The properties of the HuSaV particles have been identified mainly from the purified virus particles derived from naturally infected patients [32,33,34,35,36]. Due to limited resources, information about the virion (particularly relating to VP2) is lacking. VP2 had not yet been identified in PoSaV virions [19,23]. In the present study, we purified the HuSaV GII.3 and GI.1 particles using a large-scale cell culture and obtained >5 mg/mL of the purified virus particles, allowing us to clearly observe the VP2 protein by SDS-PAGE and to analyze the aa sequence by LC-MS/MS. Our results confirmed that VP2 is the minor structure protein associated with HuSaV particles. Interestingly, the ratios of VP1 and VP2 of the two HuSaVs were 16:1 and 20:1, which are similar to the ratio observed in FCV (15:1) [31]. Further studies are necessary to clarify the precise role(s) of VP2 in the HuSaV virion assembly.

VP1 was cleaved from the ORF1 encoded polyprotein or translated from the subgenomic RNA [37]. The cleavage site between protease-polymerase (NS6-NS7) and VP1 is E/G in HuSaV GII.2 Mc10; in addition, an N-terminal tri-peptide, MEG, of VP1 is conserved among the HuSaVs [14,15]. In the present study, we used the purified viral particles derived from HuSaV GI. 1 and GII.3 to identify the N-terminal aa sequence of the VP1, but both VP1 N-termini of HuSaV GI.1 and GII.3 were blocked, and we were unable to identify the N-terminal aa sequence of the VP1.

The density of the HuSaV GII.3 AK11 strain purified from the HuTu80 cells was 1.350 g/cm^3^, which is similar to that of HuSaV GI.1 from the stool of patients and the PoSaV Cowden strain from cells [19,35,38]. In our experiments, the empty particles were obtained from the GII.3 AK11-infected cell culture supernatants in addition to the complete virions (Figure 2b). The morphology of the empty particles is similar to that of the virus-like particles in HuSaV GI.1 Mc114, GI.2 Hou90, GII.2 Mc10, and GII.3 C12 strain produced by the expression of the VP1 gene in insect and mammalian cells [7,39,40]. However, due to the limited number of purified empty particles observed herein, we were unable to determine (i) whether VP2 is involved in the empty particles, and (ii) what the N-terminal sequence of VP1 is. It is also of interest to determine whether the empty particles are present in fecal specimens of patients during the infection.

In conclusion, we successfully produced an infectious HuSaV GII.3 strain using a reverse genetic system, and we characterized the protein component of the virion. The application of this system would be a useful method for generating infectious HuSaVs when virus-positive fecal materials are not available but the nucleotide sequence data are available. This method would also be useful for the characterization of replication as well as for studies of fundamental molecular biology, viral morphology, mechanisms of replication, antigenic analyses, and the development of vaccines for HuSaVs.

## Data Availability

The sequences of HuSaV GII.3 AK11 and GI.1 AK20 used in the study have been assigned (GenBank accession nos. LC715150 and LC715151).
